# Is Freezing of Gait in Parkinson's Disease a Result of Multiple Gait Impairments? Implications for Treatment

**DOI:** 10.1155/2012/459321

**Published:** 2012-01-12

**Authors:** Meir Plotnik, Nir Giladi, Jeffrey M. Hausdorff

**Affiliations:** ^1^Laboratory for Gait and Neurodynamics, Movement Disorders Unit, Department of Neurology, Tel-Aviv Sourasky Medical Center, Tel Aviv 64239, Israel; ^2^Department of Physiology, Sackler Faculty of Medicine, Tel-Aviv University, 69978 Tel Aviv, Israel; ^3^The Gonda Brain Research Center, Bar Ilan University, 52900 Ramat Gan, Israel; ^4^Department of Neurology, Sackler Faculty of Medicine, Tel-Aviv University, 69978 Tel Aviv, Israel; ^5^Department of Physical Therapy, Sackler Faculty of Medicine, Tel-Aviv University, 69978 Tel Aviv, Israel; ^6^Department of Medicine, Harvard Medical School, Boston, MA 02215, USA

## Abstract

Several gait impairments have been associated with freezing of gait (FOG) in patients with Parkinson's disease (PD). These include deteriorations in rhythm control, gait symmetry, bilateral coordination of gait, dynamic postural control and step scaling. We suggest that these seemingly independent gait features may have mutual interactions which, during certain circumstances, jointly drive the predisposed locomotion system into a FOG episode. This new theoretical framework is illustrated by the evaluation of the potential relationships between the so-called “sequence effect”, that is, impairments in step scaling, and gait asymmetry just prior to FOG. We further discuss what factors influence gait control to maintain functional gait. “Triggers”, for example, such as attention shifts or trajectory transitions, may precede FOG. We propose distinct categories of interventions and describe examples of existing work that support this idea: (a) interventions which aim to maintain a good level of locomotion control especially with respect to aspects related to FOG; (b) those that aim at avoiding FOG “triggers”; and (c) those that merely aim to escape from FOG once it occurs. The proposed theoretical framework sets the stage for testable hypotheses regarding the mechanisms that lead to FOG and may also lead to new treatment ideas.

## 1. Introduction

In a recent comprehensive review on the pathogenesis of freezing of gait (FOG), Nutt et al. describe several competing hypotheses that have been put forth to explain this episodic gait disturbance that mysteriously affects many, but not all patients with Parkinson's disease (PD) [1]. For example, when comparing the gait of PD patients who suffer from freezing (PD + FOG) with that of patients who do not suffer from the symptom (PD-FOG), we identified several gait properties that were abnormally altered in PD + FOG patients, even in the interictal period [2], that is, functional walking periods in between freezing episodes. We suggested that impairments in the regulation of the gait cycle (i.e., poor control of rhythmicity [3], impaired bilateral coordination of stepping [4], and increased asymmetry [5]) operate in the background, perhaps with executive function deficits, to set the stage for FOG that occurs in response to “triggering events” (e.g., turning). Some researchers highlighted the ideas that impairments in dynamic postural control while walking [6] and in step scaling [7–10] are related to the presence of FOG in PD. Other investigators underscore the importance of transitions and some suggest that visual-spatial processing is involved in the pathogenesis of FOG [11, 12]. In short, a number of different, apparently competing theories have been put forth to explain this disabling phenomenon.

Nutt et al. astutely point out that the extant hypotheses may not necessarily be exclusive [1]. In this paper, we take a closer look at this idea and show how multiple gait impairments may take place simultaneously and lead to FOG. More specifically, we propose that motor control mechanisms of two or more gait features associated with FOG may interact with each other and, under certain circumstances, deteriorate synergistically. Once the level of deterioration crosses some imaginary “redline” or critical threshold, FOG occurs.

In the following paragraphs, we introduce the theoretical framework underlying the hypothesis that synergism in the malfunction of the control of different gait features can cause an overall effect on gait performance that leads to freezing episodes in patients with PD. In particular, we describe how two seemingly independent gait features may both deteriorate when challenged, thereby increasing the propensity for FOG. We illustrate this with respect to step length scaling and gait symmetry. Then, we discuss the clinical implications of this viewpoint.

## 2. Theoretical Framework: Combined Effect of Changes in Background Levels of Gait Parameters Associated with FOG Determines the Occurrence of FOG Episode

**Figure 1:**
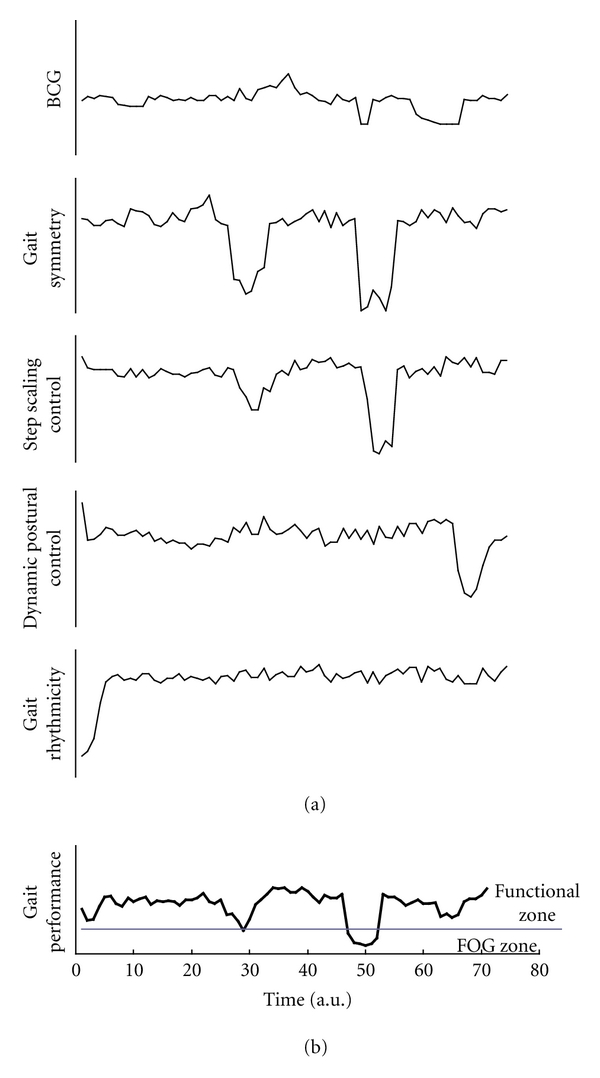
Freezing of gait and gait features deterioration. (a) Quality of performance of gait features associated with FOG (thin lines in top 5 traces) may vary over time (hypothetical data). Similarly the level of interaction between these gait features may vary with time and in response to different circumstances or provocations (see text). BCG—Bilateral coordination of gait. (b) The combination of the performances of the individual gait features dictates whether FOG will occur or whether functional walking will be maintained. If the overall performance deteriorates below a certain threshold (horizontal line), then gait freezes (FOG zone). Deterioration in the overall gait performance can be an expression of malfunction of single gait feature associated with FOG (see text). In some cases, the deterioration of one gait feature can cause the deterioration of one or more gait features as portrayed in (a). FOG—Freezing of gait, a.u.—arbitrary units

Figure 1 heuristically illustrates the concept that a FOG episode occurs when the overall gait performance is no longer sufficient to support functional gait. The overall gait performance is an expression of the combination of multiple control mechanisms; each one addresses a different aspect of walking. In Figure 1(a), the simultaneous behavior of five gait features associated with FOG in PD are depicted: (1) bilateral coordination of gait (BCG)—the control of the antiphase left-right stepping pattern; (2) gait symmetry—the control of producing similar motor program outputs to both legs, for example, equal swing times, equal step lengths (The converse of gait symmetry, gait asymmetry, GA, is a more readily measureable and can be quantified by contrasting the function of one leg with that of the other); (3) step scaling—the distance covered by each step; (4) Dynamic postural control—as expressed, for example, by center of pressure movements; and (5) Gait rhythmicity—as expressed, for example, by stride-to-stride variability (higher variability reflects lower rhythmicity).

 According to the proposed conceptual model, the individual performance in each of these domains (a) is not constant and varies over time and (b) in some instances, performance of a given feature may be influenced by another gait feature. For example, during the time period 10–20 (arbitrary units), all gait features associated with FOG maintain a fairly constant level and operate seemingly independent from each other. Similarly, when the dynamic postural control deteriorates (at time 60–70), other gait features are not influenced.

On the other hand, other gait circumstances may yield stronger dependency between different gait features. This is illustrated, for example, in the time periods 20–30 and 40–50. Deteriorations in gait symmetry are accompanied with deteriorations in step length scaling, with a suggestion that BCG may also be influenced in the 40–50 time window.

Malfunctions in gait parameters that are associated with FOG can influence the propensity for FOG not only after gait has started, but even during the preparation for walking (e.g., start hesitation that occurs prior to the initiation of walking). In these circumstances, the motor control system is not able to raise a specific gait parameter to a functional level, a fact which may also influence other gait features and result in increased propensity for FOG (lower trace in Figure 1(a)).

Theoretically, the interrelationships between different gait features and in particular gait features associated with FOG in PD can be described by the following analytical formulation:

(1) $$X_{k}\left(t\right)=\overset{n}{\underset{i=1,i\neq k}{\sum }}f_{i}\left(t,X_{i}\right),$$

where *X*_*k*_ represent any one of the gait parameters associated with FOG and *n* is the number of gait features that are associated with FOG. Equation (1) emphasizes that each gait parameter associated with FOG is influenced by any of the others, and this dependency varies with time, that is, varies with the changing “circumstances.” Furthermore, the relationship between any given pair of FOG associated gait parameters is not identical (i.e., different functions, *f*_*i*_, determine the dependence). In the case of one pair, it might be strong, while for another pair the association may be weaker.

The thick curve in Figure 1(b) represents the compound gait performance which is the combination (not necessarily linear) of all individual gait features associated with FOG. Denoting this parameter by *X*, the following analytical relationship can describe the relationship between *X* and any individual *X*_*k*_:

(2) $$X\left(t\right)=F\left(X_{1},X_{2},\ldots ,X_{n}\right).$$

According to this proposed framework, as long as the overall gait performance is maintained above a border line (i.e., a threshold), functional gait is maintained. However, once the overall gait performance deteriorates below the “threshold,” FOG occurs. As illustrated, the deterioration of gait performance and the resultant FOG may take place as a result of poor control of one or more individual gait features associated with FOG. The duration of the FOG episode is dependent upon the ability to restore the control over the gait feature(s) that deteriorates.

## 3. An Example: Do the “Sequence Effect” and Gait Asymmetry Converge?

The theoretical concept depicted above can be exemplified by probing the possibility that step scaling and gait asymmetry are related to each other and to FOG. Indeed, we suggest that insight into the pathogenesis of FOG can be gained by taking a closer look at the “sequence effect,” one of the five primary hypotheses summarized by Nutt et al. [1]. Iansek et al. first described the sequence effect and its potential contribution to FOG [8]. In a follow-up study that was designed to experimentally control the “background” step length, Chee et al. [7] found that when patients were cued to walk at a markedly reduced step length, FOG became more prevalent in subjects with PD + FOG. In this condition, FOG was associated with a progressive decrease in step length (“sequence effect”). In self-selected step length conditions, the PD + FOG subjects walked with a reduced step length as compared to PD – FOG patients. The sequence effect hypothesis posits that the progressive reduction in step length operating on a reduced background of step length leads to FOG [7].

**Figure 2:**
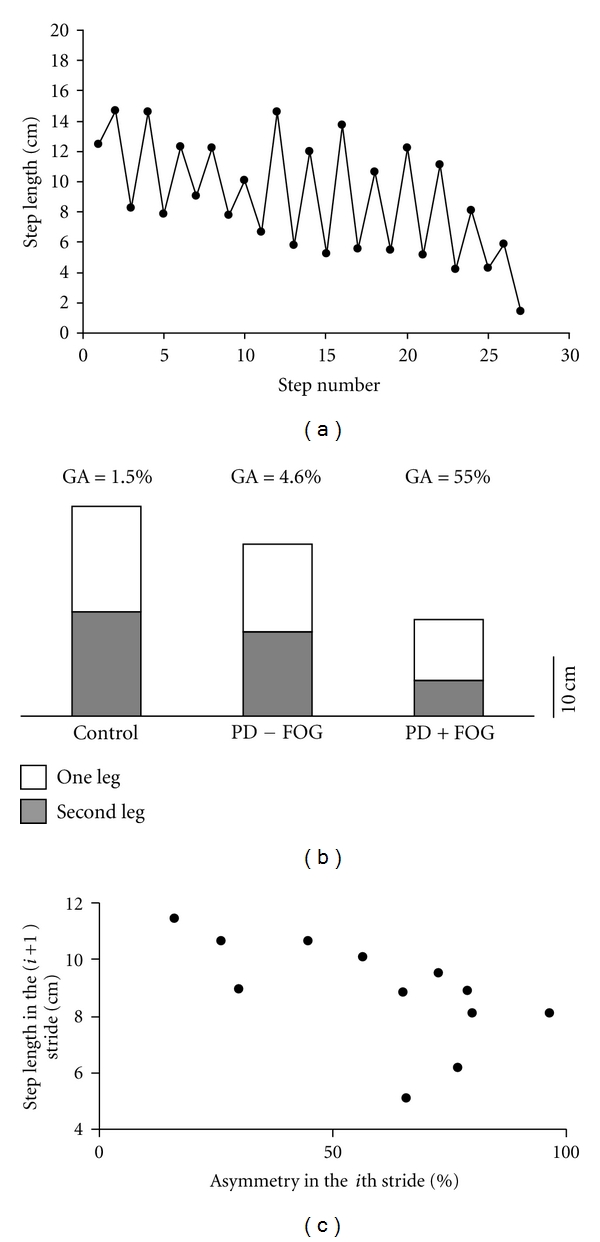
Relationship between reduced step length and asymmetry in gait. (a) Reconstruction of data presented in Figure 2 of Chee et al. [7]. The data is taken from reduced step length condition from one PD + FOG patient. (b) Similar reconstruction was performed to all 6 traces presented in Chee et al. In this panel, the mean step length value of one leg (white bar) is plotted on top of the mean step length value of the other leg (grey bar), for one reduced step length trial of PD + FOG (based on the data from (a)), a PD patient who did not experience FOG, and a control subject (based on similar reconstruction of the original traces denoted in the original figure by “b 25%01” and “c25%01,” resp.). The asymmetry coefficient for these single gait trials is depicted above. GA values indicated in the text are the means obtained from two traces from each subject. GA—Gait asymmetry as expressed by step length differences between the left and right legs. (c) Average Step Length in the (*i+*1)th stride as a function of step length asymmetry calculated for the preceding stride (*i*th). Based on data from (a).

The sequence effect was nicely illustrated by Chee et al. [7]. Data from a PD + FOG subject clearly show progressive step length reduction (see data reconstruction in Figure 2(a)). This progression is not present in the PD-FOG patient or in a healthy control subject (c.f. original figure in Chee et al.). At the same time, close inspection suggests that progressive step length reduction is also accompanied by an increased step length asymmetry. This fact jumps out from the “zigzag” pattern of the step length reduction sequence (i.e., the trace repeatedly goes up and down, with the “up” obtained from one leg the and the “down” from the other; see Figure 2(a)).

Given the putative role of asymmetry in FOG [5], we conducted growth calculations of asymmetry [5] of step length based on the data reported by Chee et al. (all six traces shown in Figure 2 of Chee et al. were included). The PD + FOG subject who displayed the sequence effect had relatively large step length asymmetry (36.2%), while the PD-FOG and the healthy control subjects who did not exhibit the sequence effect had much lower values of asymmetry (5.1% and 7.3%, resp.; see Figure 2(b) for single calculations done for single trace only). While not definitive, this finding supports the idea that more than one gait feature may be deteriorating in association with FOG. Further studies are needed to address the possibility that in reduced step length conditions, gait asymmetry increases among patients who experience FOG just prior to FOG, potentially another ingredient needed to produce the faulty state that leads to FOG.

Indeed, if we seek to develop the optimal rehabilitation program for FOG, it is critical to move beyond a description of the phenomena and to try and identify cause and effect. Examination of the data in the zigzag trace (depicted in Figure 2(a)) shows that across the strides, step length is not correlated with step length asymmetry (Spearman's *ρ* = −0.24, *P* = 0.43). There is, however, a strong inverse relationship between step length and the level of asymmetry seen in the preceding stride (Spearman's *ρ* = −0.76, *P* = 0.005): relatively increased values of asymmetry tend to *precede* relatively smaller step lengths in the next stride (Figure 2(c)). In fact, Fasano et al. [13] have recently drawn similar conclusions from their findings on gait freezing during treadmill walking with unbalanced subthalamic nucleus deep brain stimulation (STN-DBS; see below): “During poorly coordinated gait, information from the leg with the shorter stride length … might conflict with the internal cueing of the opposite leg and cause the leg with the longer stride to decrease stride length …. In PD patients, however, the strategy might further destabilize gait and induce a vicious circle of progressively shorter step length (“sequence effect”), resulting in FOG. Therefore, our findings indicate a possible link between two apparently unrelated pathogenetic theories of FOG: poor interlimb coordination and the “sequence effect” [13].

While intriguing, further work is, nonetheless, needed to determine if these findings actually reflect causality. Still, it appears that there may be more to the sequence effect than meets the eye and it may be an oversimplification to assume that a sole factor is behind the mysterious phenomenon known as FOG. This example demonstrates how multiple gait deficits, for example, asymmetry, a reduced step length, and a further decrease in the step length, are apparently simultaneously related to FOG. Acting alone, they may not always be sufficient to cause FOG. Moreover, during walking periods that are not interrupted by FOG, these gait deficits are not necessarily strongly related to each other.

Further support for this idea (i.e., that the level of interdependency between the two gait features is not constant) was obtained by revisiting data recently collected [14]. In a study in which we evaluated bilateral coordination of alternating hand tapping, we found that stride length was not significantly correlated with gait asymmetry or the phase coordination index [15] in PD patients with or without FOG (*P* > 0.422).

Turns may be another example of this principle. Turns are an activity of daily living that frequently leads to FOG [16–18]. Turns also place high demands on bilateral coordination. In addition, during a turn, step length reduction may be exacerbated due to the need to reduce the step length of the inner leg. The two effects, high demand on bilateral coordination and reduced step length, may superimpose to cause FOG. These and additional interfering effects such as attention loading should be studied in light of the possibility that they work synergistically.

## 4. Clinical Implications: How to Address the Multifactorial Aspects of Freezing of Gait?

Treating freezing of gait in PD is very complicated and to the best of our knowledge no “magic bullet” has yet been identified. On the other hand, a conceptual approach acknowledging that synergy and multiple influences between gait control mechanisms have an impact on FOG may enable researchers to generate some new thinking about treatment opportunities as well as mechanisms. This raises an interesting, practical question: what should be the targets of treatments designed to reduce FOG?

**Figure 3:**
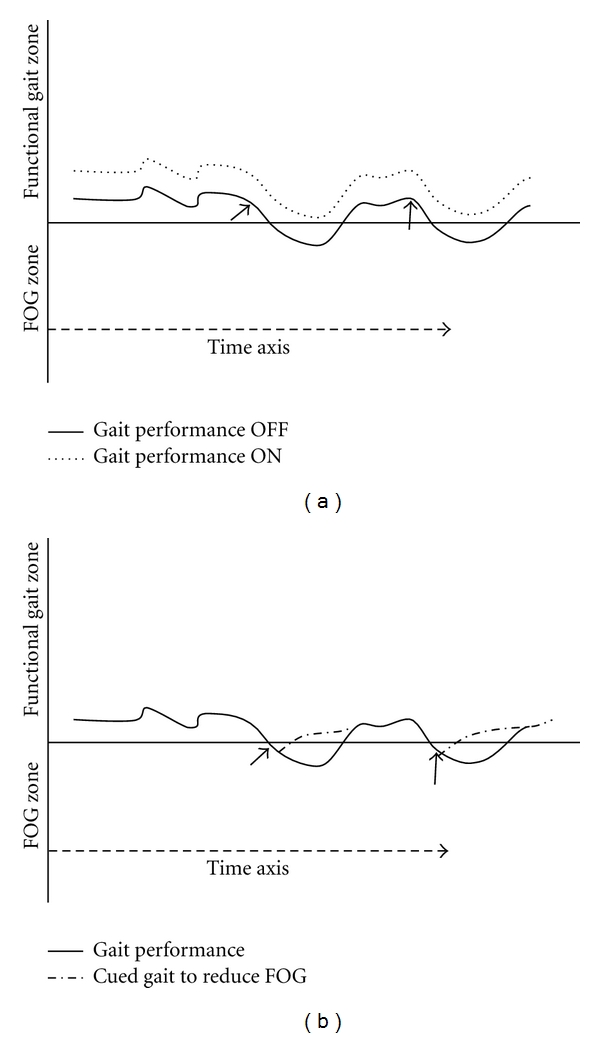
Intervening to improve overall gait performance and to reduce the FOG burden. (a) Improving gait performance in general, for example, by maintaining a sustained effective therapeutic effect on multiple gait features associated with FOG (recall Figure 1(a)) is a target for therapy that will likely reduce the FOG burden (see text). The black arrows reflect two instances where FOG might normally occur when the patient is OFF anti-parkinsonian medications. This might represent some diversion or divided attention that increase the likelihood of FOG. In the ON medication condition, when the overall performance is further away from the FOG threshold (horizontal line), attention still has a negative effect, but it is no longer sufficient to cause FOG. In general, one way of reducing the likelihood of FOG is to move the overall gait performance further away from this threshold. (b) Online intervention may reduce the duration of FOG episodes (see text). Key: Gait performance OFF—gait during the “off” medication periods. Gait performance ON—gait during the “on” medication periods.

Keeping in mind the theoretical idea that the overall gait performance and the propensity to FOG is the product of a combination of individual gait features associated with FOG (Figure 1(b)), we turn now to discuss what may affect the individual gait features in a way that the compound gait performance moves from the functional zone to the FOG zone.  Figure 3(a) illustrates two instances (“triggers”) that “push” gait from the functional zone to the FOG zone (denoted by black arrows). This might reflect one of many triggers such as dividing attention while walking, something that has been associated with FOG [19–21]. When a patient's focus of attention is diverted from walking, the likelihood for freezing will increase since the attention demanding task is being dealt with at the expense of gait control. In fact, all of the five gait features associated with FOG that were described in Figure 1(a) have been shown to deteriorate when subjects with PD perform a dual task [22–25]. It is important to note that triggers of FOG are likely to be less effective if the baseline overall performance of gait is enhanced (in Figure 3(a), compare the dotted trace to solid trace). This fact is supported by the observation that freezing episodes are less frequent during the “ON” phase of the medication cycle as compared to the “OFF” phase [16]; in the “ON” phase, many features of gait improve, enhancing overall gait performance.

Taking these considerations into account it, seems that interventions for the rehabilitation/treatment of FOG should address one or more of the following aspects: (a) improving the overall, background gait performance, in particular gait features associated with FOG, in order to perform within the “envelope” of the functional gait zone; (b) improving the response to the occurrence of FOG provoking triggers; (c) minimizing the impact of freezing on gait regulation. Below is a brief review of recently proposed therapeutic approaches addressing these elements.

### 4.1. Improving Baseline Gait Performance to Reduce FOG Propensity

Fasano et al. [13] took advantage of the fact that in patients with bilateral implementation of electrodes for STN-DBS asymmetric stimulation of the subthalamic nuclei can result in modulation of the symmetry and coordination between legs. They examined the gait of subjects with PD who suffer from the FOG symptom and showed that uneven stimulation (stimulation voltage decrease in the better functioning brain side) improved BCG as compared to the regular prescribed stimulation (by about 60%). The frequency of FOG episodes decreased 10-fold and their duration decreased more than 20-fold. Additional gait features (e.g., cadence and stride length) improved as well. This study illustrates how improving baseline performance of gait feature associated with FOG (i.e., BCG and stride length) can result in a significant reduction in FOG.

Physiotherapy interventions may also be effective in changing usual-walking gait parameters. For example, treadmill training can be beneficial in increasing stride length, but not cadence (i.e., rhythmicity) [26]. However, the long-term carry-over effects of treadmill interventions still remain to be seen.

### 4.2. Targeting the Triggers for FOG

From a theoretical point of view, there are two potential types of triggers that can drive the gait control system to such poor management that FOG will more likely occur. The first one is attention shifts (already mentioned above). It is, therefore, reasonable to assume that an intervention that trains the subject to improve his/her performance in dual tasking conditions will result in the reduction of the FOG burden. To the best of our knowledge, only a few studies administrated dual-task (DT) based intervention in subjects with PD. Results published so far are supportive of this notion. For example, Canning et al. [27] and Yogev-Seligmann et al. [28] found improvements in gait speed in response to DT training which was maintained in the retention phase. Other investigators observed improvements in stride length [29]. If training improves DT gait speed and stride length, overall performance is likely to move away from the FOG threshold.

The second type of FOG triggering circumstances are related to transitions between walking trajectory types. These triggers which may shift the locomotion control to the FOG zone (e.g., the transition from straight line walking to turning) are a reasonable goal for intervention. Spatial circumstances that challenge one or more of the gait features associated with FOG can cause malfunction in that gait feature that will lead to overall deterioration (i.e., “trajectory triggers”). For example, changing trajectories from straight line walking to turning poses high demands on BCG since gait control now produces different motor programs to the axial (inner) and the pivotal (outer) leg; this mismatch challenges coordination. In addition, the step length reduction seen in the inner leg challenges the step scaling control and may trigger the sequence effect. Likewise, walking through narrow passages may lead to slowness of gait and reduction in step length since choosing a leading leg for passing through the passage is both an attention and coordination demanding situation.

If effective training results in adaptation of the motor system to rapid accommodation of the post-transition gait task, then the transitioning effect (i.e., between two gait patterns) may have lesser impact on gait. In a pilot study, Hong and Earhart [30] used a rotating treadmill to extensively expose subjects with PD who suffer from FOG to circular walking. Following this training, the two subjects who participated in the study exhibited substantial improvement and immediate reduction in freezing episodes. The authors suggested that after “practice of externally cued turning, a motor pattern appropriate for turning may become more automatic, facilitating the ease of switching between straight walking and turning. This may be the mechanism of improved turning ability and reduced freezing following rotating treadmill training” [30]. Perhaps, carefully designed gait training programs for particular conditions (e.g., narrow passages) will improve the response of the patient to changing gait patterns required spatial circumstances that would otherwise impact on multiple gait features and likely provoke FOG.

### 4.3. Assistive Device for Alleviating the FOG Symptom

In recent years, wearable mobility measures were used in studies that documented locomotion patterns in patients with PD [31]. The compound gait performance which sustained functional gait or “falls” into the FOG zone (Figures 1 and 3) can be a subject of quantification. This quantification may be achieved by the analysis of data recorded by mobility measures. If such quantification can take place in real time, it might be possible to identify gait deterioration into the FOG zone. Then, an automated response in the form of external cue can be elicited to help the patient to restore functional gait. This concept is heuristically illustrated in Figure 3(b). The two arrows point to the places in which gait performance cross the negative threshold into the FOG zone. If efficient automatic detection device will identify these points and elicit external cue, then the subject utilizing the information from the external cueing will restore functional gait (dashed lines departing from the solid line) more rapidly. A good “candidate” for effective external cue is the rhythmic auditory stimulation which has been proven effective in improving gait in subjects with PD (for review Lim et al. [32]).

In a pilot study, we demonstrated the feasibility of this strategy [33, 34]. Within a 3-second delay, a wearable device based on set of accelerometers identified in real time more than 200 FOG episodes with technical sensitivity >70% and specificity of >80%. These promising results open the venue for assistive devices to ameliorate the FOG symptom, rather than to treat it.

## 5. Summary

This paper addresses the problem of FOG in PD from a slightly different angle. The conceptual framework states that more than one gait control mechanism may be impaired in association with FOG and that the control of gait features associated with FOG may, under certain circumstances or triggers of FOG, interact. Paradoxically, this rather complex nature of the potential pathogenesis of FOG in PD motivates pursuing more than one scheme of intervention with several degrees of freedom. While curing the symptom seems out of reach at the moment, recent findings support the promise that sooner rather than later, the symptom will be curtailed during the daily living of patients with PD.

## References

[1] Nutt JG, Bloem BR, Giladi N, Hallett M, Horak FB, Nieuwboer A (2011). Freezing of gait: moving forward on a mysterious clinical phenomenon. *The Lancet Neurology*.

[2] Plotnik M, Hausdorff JM (2008). The role of gait rhythmicity and bilateral coordination of stepping in the pathophysiology of freezing of gait in Parkinson’s disease. *Movement Disorders*.

[3] Hausdorff JM, Schaafsma JD, Balash Y, Bartels AL, Gurevich T, Giladi N (2003). Impaired regulation of stride variability in Parkinson’s disease subjects with freezing of gait. *Experimental Brain Research*.

[4] Plotnik M, Giladi N, Hausdorff JM (2008). Bilateral coordination of walking and freezing of gait in Parkinson’s disease. *European Journal of Neuroscience*.

[5] Plotnik M, Giladi N, Balash Y, Peretz C, Hausdorff JM (2005). Is freezing of gait in Parkinson’s disease related to asymmetric motor function?. *Annals of Neurology*.

[6] Jacobs JV, Nutt JG, Carlson-Kuhta P, Stephens M, Horak FB (2009). Knee trembling during freezing of gait represents multiple anticipatory postural adjustments. *Experimental Neurology*.

[7] Chee R, Murphy A, Danoudis M, Georgiou-Karistianis N, Iansek R (2009). Gait freezing in Parkinsons disease and the stride length sequence effect interaction. *Brain*.

[8] Iansek R, Huxham F, McGinley J (2006). The sequence effect and gait festination in parkinson disease: contributors to freezing of gait?. *Movement Disorders*.

[9] Nieuwboer A, Dom R, De Weerdt W, Desloovere K, Fieuws S, Broens-Kaucsik E (2001). Abnormalities of the spatiotemporal characteristics of Gait at the onset of freezing in Parkinson’s disease. *Movement Disorders*.

[10] Nieuwboer A, Dom R, De Weerdt W, Desloovere K, Janssens L, Stijn V (2004). Electromyographic profiles of gait prior to onset of freezing episodes in patients with Parkinson’s disease. *Brain*.

[11] Cowie D, Limousin P, Peters A, Day BL (2010). Insights into the neural control of locomotion from walking through doorways in Parkinson’s disease. *Neuropsychologia*.

[12] Lebold CA, Almeida QJ (2010). Evaluating the contributions of dynamic flow to freezing of gait in parkinson’s disease. *Parkinson’s Disease*.

[13] Fasano A, Herzog J, Seifert E (2011). Modulation of gait coordination by subthalamic stimulation improves freezing of gait. *Movement Disorders*.

[14] Plotnik M, Herman T, Shaviv E, Brozgol M, Giladi N, Hausdorff JM (2009). Impaired bilateral coordination of gait and upper extremity rhythmic movements in Parkinson's disease: association with freezing of gait. *Movement Disorders*.

[15] Plotnik M, Giladi N, Hausdorff JM (2007). A new measure for quantifying the bilateral coordination of human gait: effects of aging and Parkinson’s disease. *Experimental Brain Research*.

[16] Schaafsma JD, Balash Y, Gurevich T, Bartels AL, Hausdorff JM, Giladi N (2003). Characterization of freezing of gait subtypes and the response of each to levodopa in Parkinson’s disease. *European Journal of Neurology*.

[17] Snijders AH, Nijkrake MJ, Bakker M, Munneke M, Wind C, Bloem BR (2008). Clinimetrics of freezing of gait. *Movement Disorders*.

[18] Spildooren J, Vercruysse S, Desloovere K, Vandenberghe W, Kerckhofs E, Nieuwboer A (2010). Freezing of gait in Parkinson’s disease: the impact of dual-tasking and turning. *Movement Disorders*.

[19] Amboni M, Cozzolino A, Longo K, Picillo M, Barone P (2008). Freezing of gait and executive functions in patients with Parkinson’s disease. *Movement Disorders*.

[20] Camicioli R, Oken BS, Sexton G, Kaye JA, Nutt JG (1998). Verbal fluency task affects gait in Parkinson’s disease with motor freezing. *Journal of Geriatric Psychiatry and Neurology*.

[21] Naismith SL, Shine JM, Lewis SJG (2010). The specific contributions of set-shifting to freezing of gait in Parkinson’s disease. *Movement Disorders*.

[22] Allen RM, Carlson-Kuhta P, Jacobs JV, Nutt JG, Horak FB (2009). Effect of cognitive dual-task on balance in Parkinson's disease. *Movement Disorders*.

[23] Plotnik M, Giladi N, Hausdorff JM (2009). Bilateral coordination of gait and Parkinson’s disease: the effects of dual tasking. *Journal of Neurology, Neurosurgery and Psychiatry*.

[24] Yogev G, Giladi N, Peretz C, Springer S, Simon ES, Hausdorff JM (2005). Dual tasking, gait rhythmicity, and Parkinson’s disease: which aspects of gait are attention demanding?. *European Journal of Neuroscience*.

[25] Yogev G, Plotnik M, Peretz C, Giladi N, Hausdorff JM (2007). Gait asymmetry in patients with Parkinson’s disease and elderly fallers: when does the bilateral coordination of gait require attention?. *Experimental Brain Research*.

[26] Mehrholz J, Friis R, Kugler J, Twork S, Storch A, Pohl M (2010). Treadmill training for patients with Parkinson’s disease. *Cochrane Database of Systematic Reviews*.

[27] Canning CG, Ada L, Woodhouse E (2008). Multiple-task walking training in people with mild to moderate Parkinson’s disease: a pilot study. *Clinical Rehabilitation*.

[28] Yogev-Seligmann G, Giladi N, Brozgol M, Hausdorff JM A training program to improve gait while dual tasking in patients with parkinson's disease: a pilot study.

[29] Brauer SG, Morris ME (2010). Can people with Parkinson’s disease improve dual tasking when walking?. *Gait and Posture*.

[30] Hong M, Earhart GM (2008). Rotating treadmill training reduces freezing in Parkinson disease: preliminary observations. *Parkinsonism and Related Disorders*.

[31] Moore ST, MacDougall HG, Ondo WG (2008). Ambulatory monitoring of freezing of gait in Parkinson’s disease. *Journal of Neuroscience Methods*.

[32] Lim I, van Wegen E, de Goede C (2005). Effects of external rhythmical cueing on gait in patients with Parkinson’s disease: a systematic review. *Clinical Rehabilitation*.

[33] Bächlin M, Plotnik M, Roggen D, Giladi N, Hausdorff JM, Tröster G (2010). A wearable system to assist walking of parkinsońs disease patients benefits and challenges of context-triggered acoustic cueing. *Methods of Information in Medicine*.

[34] Bachlin M, Plotnik M, Roggen D (2010). Wearable assistant for Parkinsons disease patients with the freezing of gait symptom. *IEEE Transactions on Information Technology in Biomedicine*.

